# *Campylobacter* infection in a cohort of rural children in Moramanga, Madagascar

**DOI:** 10.1186/1471-2334-14-372

**Published:** 2014-07-05

**Authors:** Rindra Vatosoa Randremanana, Frédérique Randrianirina, Philippe Sabatier, Hanitra Clara Rakotonirina, Arthur Randriamanantena, Iony Manitra Razanajatovo, Rila Ratovoson, Vincent Richard

**Affiliations:** 1 Unité Epidémiologie - Institut Pasteur de Madagascar, BP 1274, Antananarivo 101, Madagascar; 2 Centre de Biologie Clinique - Institut Pasteur de Madagascar, BP 1274, Antananarivo 101, Madagascar; 3 Equipe Environnement et Prédiction de la Santé des Populations- TIMC-IMAG, UMR 5525, CNRS-UJF- VetAgroSup, 1, avenue Bourgelat, F 69280 Marcy l’Etoile, Lyon, France; 4 Laboratoire d’Epidémio-surveillance-Institut Pasteur de Madagascar, BP 1274, Antananarivo 101, Madagascar; 5 Unité Epidémiologie - Institut Pasteur de Dakar, Dakar, Sénégal

**Keywords:** *Campylobacter* infection, Cohort study, Diarrhoea, Rural area, Madagascar

## Abstract

**Background:**

*Campylobacter* infection is the most common cause of bacterial gastroenteritis in developing countries, including Madagascar. Reports of pathogenicity have not been consistent and repeated exposures over time seem to lead to the development of protective immunity in developing areas. We conducted this study to support evidence for these hypotheses by exploring the association between infection and age, the reoccurrence of infection and the pathogenicity of *Campylobacter*.

**Methods:**

We carried out a community-based longitudinal study of children under the age of 24 months in two rural villages in Moramanga, Madagascar. Children were visited twice a week and a stool specimen was collected in cases of diarrhoea. Stools specimens were collected bimonthly from all children enrolled, regardless of symptoms. Children were followed-up until the age of 36 months.

**Results:**

Between January 2010 and May 31^st^ 2012, 508 children were included in the cohort. We detected 319 episodes of *Campylobacter* infection in total, and 43.3% (*n* = 220) of the children had at least one episode of intestinal *Campylobacter* infection. The rate of *Campylobacter* isolation from stool specimens was 9.3%. The annual incidence rate for symptomatic *Campylobacter* infection was 0.05 episodes/child. The probability of *Campylobacter* infection was highest between the ages of six and 23 months. Taking children under six months of age as the reference group, the age-specific odds ratio for the association was 5.0 (95% CI: 2.9-8.6) for children aged six to 11 months, 5.7 (95% CI: 3.3-10.0) for children aged 12 to 17 months and 3.3 (95% CI: 1.8-5.8) for children aged 18 to 23 months. A second episode of infection occurred 63 days after the first episode in children with primary infections, and after 137 days in children with multiple infections (*p* < 0.01). First episodes of *Campylobacter* infection were associated with diarrhoea (odds ratio = 16.1; 95% CI: 1.8-140.8).

**Conclusion:**

Our findings suggest that protective immunity to *Campylobacter* may be acquired over time, following repeated exposures. However, *Campylobacter* infection prevention measures should be reinforced in the first year of life, as this age seems to be associated with the highest risk of diarrhoea during *Campylobacter* infection.

## Background

Although, diarrhoea-related mortality is decreasing worldwide since the introduction of oral rehydration therapy, it remains high. In Africa, the estimated annual reduction of diarrhoeal deaths since 2000 was about 4% per year [1]. In total 3.6 million children each year die before reaching their fifth birthday in Africa, and diarrhoeal disease accounts for 11% of these deaths [1]. Disease morbidity and the incidence of diarrhoea in particular, are declining more modestly [2]. The importance of pathogens such as rotavirus and *Escherichia coli* in the aetiology of severe childhood diarrhoea in developing countries is well recognised [3,4]. However the role of *Campylobacter* is not well understood either in the community setting or in hospitalised subjects and outpatients.

The epidemiological features of *Campylobacter* infection differ between developed and developing countries. *Campylobacter* is endemic to developing countries, where it is, one of the most frequently isolated bacteria from both diarrhoeic and non-diarrhoeic children [5]. Current estimates of the proportion of diarrhoea cases attributable to *Campylobacter* infection are derived from a small number of studies, but it is believed to be high, between 5 and 20% [5]. A case-control study for diarrhoea was conducted in 14 districts of Madagascar in 2008 in children under-five, by the Pasteur Institute of Madagascar [6]. Its findings suggested that *Campylobacter* was the second most frequently isolated enteropathogen, after parasites, in children under the age of five years, with a prevalence of 9.7%. However, its frequency did not differ between cases and control subjects.

Many studies have suggested that the development of protective immunity in children from developing countries may account for the high rates of asymptomatic *Campylobacter* infection and for the decrease in the proportion of infected subjects presenting illness with increasing age [7,8]. *Campylobacter* isolation rates are highest during the first two years of life and appear to decline with age [5,9]. The development of immunity might also affect the recurrence of infection. Immunity following a first episode of campylobacteriosis might decrease the risk of subsequent events, which might also vary over time and with patient characteristics [10].

In 2008, the cross-sectional study [6] conducted in Malagasy children under-five have not found an association between *Campylobacter* infection and occurrence of diarrhoea. Thus, we investigated the pathogenicity of *Campylobacter* through a cohort study and assessed the hypothesis of the development of immunity after *Campylobacter* infection in Madagascar. We conducted the study in young children under the age of 36 months, known as the age at higher risk of *Campylobacter* infection.

## Methods

### Setting and study population

This study was conducted in the low-income rural areas of Befotsy and Ampitambe, Moramanga, in the middle of the eastern region of Madagascar. These two villages were the pilot areas of the Health and Demographic Surveillance Site of Moramanga (HDSS Moramanga) and were investigated during the case-control study carried out in 2008 [6]. The highest prevalence of *Campylobacter* infection in children with diarrhoea was found in the district of Moramanga (20.7%). The prevalence of *Campylobacter* was 20% in Befotsy and 10% in Ampitambe. The local population is 4231 inhabitants, most of whom are engaged in agricultural activities. The 1006 households lack basic sanitary facilities, use water from the river or a traditional well for drinking and often have free-range domestic chickens at home; the risk of faecal contamination of the environment is therefore likely to be high.

### Cohort enrolment

We used data from HDSS Moramanga, in which longitudinal demographic surveillance was carried out on the population of four communities in the district of Moramanga. The HDSS Moramanga conducted a door-to-door census, and collected demographic data and data for each individual household in the two villages at a given time point (water supply, goods, ownership of animals, etc…). Children under the age of 24 months living in either of the two villages were eligible for enrolment in this study. An open cohort of children enrolled before the age of 24 months was followed up from January 2010 to May 2012. All the children were monitored until the age of 36 months. Children who moved within the study area were followed up at their new homes, whereas those who moved outside the study area were withdrawn from the study. On enrolment, an interview was conducted with one of the child’s parents, to obtain information about the child, including breastfeeding and nutritional status. Each of the children enrolled provided a stool specimen for the isolation of Campylobacter spp. Enrolment continued throughout the study, for new infants born into a household and for young children moving into the village.

### Surveillance activities

#### Twice a week diarrhoeal surveillance

From the day of enrolment in the study until 36 months of age, a study physician and a locally recruited community health worker (CHW) visited each of the children twice a week at their homes. The children’s mothers were asked about any episodes of diarrhoea since the last visit. If a child had diarrhoeal illness, the physician carried out clinical and anthropometric examinations, collected a stool sample for *Campylobacter* culture and provided oral rehydration therapy and/or other treatment if indicated. Treatment was given in accordance with Ministry of Health guidelines. Antimicrobial treatment was provided if diarrhoea was associated with fever or blood/mucus in the stools. A pictorial diarrhoea diary was given to the mothers of children with diarrhoea, for recording the number of bowel movements and the consistency of the faeces, from the first day of diarrhoea until its cessation. Movements of children to areas outside the study area, deaths and other losses to follow-up were recorded by the CHW during the twice a week visits.

#### Bimonthly surveillance

We also carried out a cross-sectional surveillance. All children included in the cohort, regardless of their history of diarrhoea, were surveyed once every two months (60 days), with the collection of a stool specimen for *Campylobacter* isolation. In these surveys, weight and length/height data were also collected. This cross-sectional study provided us with data on asymptomatic *Campylobacter* infection.

These surveillance activities were performed for the purposes of this study only.

### Definitions

– *A day with diarrhoea* was defined as a 24-hour period in which three or more loose stools taking the form of the container or any number of stools containing blood were obtained [11]. For small, exclusively breastfed children, if the stools were not bloody, diarrhoea was defined as an increase in the frequency or a reduction of the consistency of the stools with respect to what the mother considered normal for her child.

– *Episodes of diarrhoeal illness* were defined using a minimum three-day diarrhoea-free gap to mark the beginning of a new episode.

– *Campylobacter* infection was defined as symptomatic if *Campylobacter* was isolated from diarrhoeal stools or within a period of five days before and after an episode of diarrhoea.

– *Campylobacter* infection was defined as asymptomatic if there were at least five consecutive symptom-free days before and after the isolation of *Campylobacter* from faeces.

– The days at risk included the three-day period following the occurrence of an episode of diarrhoea.

– Primary *Campylobacter* infection was defined as the first isolation of *Campylobacter* from a stool specimen collected during enrolment, or at the twice a week or bimonthly surveillance visits, from a child enrolled within 28 days of birth.

– Multiple *Campylobacter* infections were defined as the isolation of *Campylobacter* from a stool specimen collected during enrolment, or at the twice a week or bimonthly surveillance visits, from a child enrolled after the age of 28 days.

### Microbiological analyses

For the isolation of Campylobacter spp*.*, we cultured fresh faecal specimens from children with and without diarrhoea directly in the field on selective agar plates (Karmali). Plates were incubated at 37°C under microaerophilic conditions (Campygen, Oxoid France) for 48 to 72 hours. The identification of *Campylobacter* isolates was confirmed with the Campy dry spot kit, a haemagglutination test from Oxoid (England), as recommended by the manufacturer. We differentiated between *Campylobacter jejuni (C. jejuni), Campylobacter coli (C. coli)* and other species by the multiplex polymerase chain reaction (PCR) method [12]. This differentiation was conducted on 271 samples of *Campylobacter* isolates selected at random.

### Data analysis

We analysed data from 19^th^ January 2010 until 31^st^ May 2012. Person-time at risk was calculated as the observed number of days at risk between episodes. Incidence rates were calculated by dividing the number of episodes by the number of child-years of observation. We estimated the incidence rates for diarrhoea and symptomatic infection. Children were assigned to age groups: 0 to 5 months, 6 to 11 months, 12 to 17 months, 18 to 23 months, 24 to 29 months and 30 to 36 months. This classification of age group was chosen in accordance with our hypothesis that exposure and infection risk vary with age.

Anthropometric measurements were transformed into weight-for-height/length, height/length-for-age and weight-for-age Z scores based on WHO standard reference population. Stunting, wasting and underweight were defined as height/length-for-age, weight-for-height/length and weight-for-age less than -2 standard-deviations from the reference standards for children of the same age and sex, respectively [13].

### Association between infection and age

#### At the time of enrolment

The Chi-squared test was used to measure associations between *Campylobacter* infection and age group at the time of enrolment. If a significant result was obtained by the Chi-squared test, we assessed the strength of association between *Campylobacter* infection and age group at the time of enrolment by logistic regression. The unit of analysis was the individual child. Data collected at the time of enrolment were used, with age group considered as the explanatory variable and the infection status of the faecal specimen as the outcome.

#### During follow-up

During follow-up, the association of *Campylobacter* infection and symptomatic *Campylobacter* infection with age group was assessed with a logistic mixed regression model. The outcome variable was *Campylobacter* infection or symptomatic *Campylobacter* infection status, and age group was the main explanatory variable. For the two analyses, we incorporated socioeconomic and household data collected at the time of enrolment, nutritional status data obtained during enrolment and follow-up as potential confounding variables. We included in the mixed models all potential confounding variables with a *p*-value <0.2 in the univariate analyses. We then used backward elimination to identify confounding variables for inclusion in the final model. A Wald Chi-squared test was used to assess the significance of each of the variables tested and odds ratios were calculated to quantify their effects. The follow-up visits for a given child were not statistically independent entities. We therefore took into account the correlation between repeated observations for each individual, between multiple children living in the same household and in the same village. Thus, for the two mixed models, we added each observation for each child, household and village as a random effect. The use of a random effect made it possible to adjust for the correlation between data obtained from the same individual, the same household or the same village and to measure the variability between these data.

The unit of analysis was the individual visit, with the collection of a stool sample, for investigations of the relationship between *Campylobacter* infection and age. For analysis of the relationship between symptomatic *Campylobacter* infection and age, we considered each visit at which a diarrhoeal stool sample was obtained (diarrhoeal episode) as the unit of analysis. We took the 0-5 months age group as the reference group.

Data for *Campylobacter* infection status (overall infection, symptomatic infection) were obtained at the time of enrolment, and at the twice a week and bimonthly surveillance visits.

### Pathogenicity of Campylobacter

The association between *Campylobacter* infection and diarrhoeal episodes was investigated with logistic regression models. This analysis was performed on newborns enrolled within 28 days of birth. Children with diarrhoea were considered as the outcome, with *Campylobacter* infection as the explanatory variable.

### Recurrent episodes

Survival analysis was used to assess the time to recurrent episodes of *Campylobacter* in children with primary infection and those with multiple infection. We used log-rank tests to compare the two Kaplan-Meier survival curves. We used Cox proportional hazard models to assess the extent to which the timing of recurrent episodes could be explained by potential confounders. Time to event was estimated as the interval between the first and second episodes of infection.

Data were analysed with R software version 2.12.1 (R Development Core Team (2007). For all statistical tests, a *p*-value below 0.05 was considered statistically significant.

### Ethics

Written informed consent was obtained from the parent or guardian of each child before enrolment. The study was approved by the National Ethics Committee of the Ministry of Health of Madagascar (Number 002-CE/MINSAN – 01/13/2012).

## Results

We recruited 210 children at the start of the study. Thereafter, 68 new births, 54 immigrants to the study and 176 children included after the age of 28 days were enrolled. The mean age of children at the start of the study was 11.7 months (standard deviation: 7.4; range: 0.2-23 months; median: 11.6 months). During the observation period, four children died from causes other than diarrhoea during their first year of life and 68 (13.4%) dropped out of the study due to a withdrawal of consent (22/508) or migration out of the study area (46/508). The study population comprised 508 children, who were followed for a total of 256,366 child-days (702.4 child-years). The median follow-up time was 505.7 days (interquartile range [IQR]: 373 days). The cohort of 508 children consisted of 259 girls and 249 boys (male/female ratio = 0.9). The characteristics of the cohort children were shown in Table 1.

**Table 1:** **Characteristics of the cohort children during a 28-month longitudinal community study of *Campylobacter *** **infection, Moramanga, 2010-2012**

**Characteristics**		**N**	**(%)**
**Demographic data n = 508**			
Sex	Male	249	(49.0)
	Female	259	(51.0)
Age group (months)	< 6	295	(58.1)
	6-11	74	(14.5)
	12-17	64	(12.6)
	18-23	75	(14.7)
**Nutritional status data n = 506**			
Stunting	Yes	151	(29.8)
	No	355	(70.2)
Underweight	Yes	46	(9.1)
	No	460	(81.9)
Wasting	Yes	5	(0.9)
	No	501	(99.1)
**Households data n = 439**			
Number of persons/room (mean ± SD) 3.7 (±1.7)			
Floor	Concrete	271	(67.1)
	Mud	168	(32.9)
Cooking area	Yes	411	(80.9)
	No	28	(19.1)
Shower area	Yes	50	(11.4)
	No	389	(88.6)
Latrin	Yes	281	(64.0)
	No	158	(36.0)
Domestic animals	Yes	134	(30.5)
	No	305	(69.5)
Livestock #	Yes	109	(24.8)
	No	330	(75.2)
Fowl§	Yes	279	(63.5)
	No	160	(36.5)
Goods£	Yes	434	(98.8)
	No	5	(1.2)
Source of drinking water	Tubewell, borehole	201	(45.8)
	Surface waters	239	(54.2)
Water drinking storage	Protected	139	(31.6)
	Unprotected	300	(68.4)
Garbage in the concession	Yes	222	(50.6)
	No	217	(49.4)
**Data about mother n = 416**			
Age (years) (mean ± SD) 28.3 (±9)			
Maternal education	Primary	294	(70.7)
	Secondary	91	(21.9)
	Superior	4	(0.9)
	No education	27	(6.5)

SD: standard-deviation.

#Ownership of one of the following: cow, ox, sheep, pig, rabbit.

§Ownership of one of the following: chicken, duck, goose.

£Ownership of one of the following: radio, television, bicycle, sewing machine, mobile phone, rice fields, house.

Over the two 2-year study period, 3424 stool samples were tested for *Campylobacter*. In total, 2965 (87%) of these samples were collected from children without diarrhoea during the cross-sectional survey, which was conducted at two-month intervals. The rate of *Campylobacter* isolation from all samples was 9.3% (319/3424): 8.9% (41/459) from diarrhoeic samples and 9.4% (278/2965) in non-diarrhoeic samples. Overall, 43.3% (220/508) of children had at least one *Campylobacter* infection; 16.4% (36/220) of the infected children had diarrhoea and 32.3% (71/220) had more than one episode of *Campylobacter* infection. Overall, 13.9% (71/508) of all children had more than one episode of infection. Identification to the species level was carried out for 271 of the 319 *Campylobacter* isolates: 190 (70.1%) were *C. jejuni*, 64 (23.6%) were *C. coli* and 17 (6.3%) belonged to other species. There were no statistically significant difference between the isolation rate of *C. jejuni* and *C. coli* from diarrhoeal and non-diarrhoeal samples (p = 0.16, Chi-squared test). We found that children infected with *C. jejuni* were likely to have subsequent infection than those infected with *C.coli* (p = 0.002, Chi-squared test): 4.7% (3/64) of children infected with *C. coli* and 21.6% (39/180) of those infected with *C. jejuni* had subsequent infection*. Campylobacter* isolation rates from diarrhoeic and non-diarrhoeic samples, by age group, are shown in Table 2.

**Table 2:** **Rate of isolation of enteric *Campylobacter *** **from diarrhoeic and non-diarrhoeic samples, Moramanga, 2010-2012**

		**Number of positive samples**				
		**Diarrhoeal stools**		**Non-diarrhoeal stools**		
**Age group**	**Child-days at risk**	**Number of episodes**	**Number positive (%)**	**Number of tests**	**Number positive (%)**	**Number of infections**
0-5 months	27,616	64	4 (6.2)	564	14 (2.5)	18
6-11 months	77,208	171	16 (9.3)	641	93 (14.5)	109
12-17 months	68,184	102	10 (9.8)	557	90 (16.1)	100
18-23 months	25,146	64	6 (9.4)	535	46 (8.6)	52
24-29 months	30,859	40	4 (10.0)	399	22 (5.5)	26
30-36 months	27,353	18	1 (5.5)	269	13 (4.8)	14
**Total**	**256,366**	**459**	**41 (8.9)**	**2965**	**278 (9.4)**	**319**

At the time of enrolment, 6.3% (*n* = 32) of the children were already infected with *Campylobacter*: 6.2% (2/32) were symptomatic and the remaining (30/32) children were asymptomatic. The prevalence of *Campylobacter* infection, by age group, at the time of enrolment was 4.6% (17/369) for children under 12 months of age and 10.8% (15/139) for children aged 12 months or older. The frequency of *Campylobacter* infection at the time of enrolment differed significantly between age groups (Chi-squared test, *p* < 0.001). The age-specific odds ratios for *Campylobacter* infection at the time of enrolment were 8.6 (95% confidence interval [CI]: 3.0-24.1), 5.9 (95% CI:1.9-18.3), 5.7 (1.9-17.2) for the 6-11 month, 12-17 month and 18-23 month age groups, respectively.

The probability of infection during follow-up varied with age. In the logistic mixed model, taking children under six months of age as the reference group, the odds of being infected with *Campylobacter* were five times higher in children aged between six and 18 months, the odds ratio were 5 (95% CI: 2.9-8.6) for children aged six to 11 months and 5.7 (95% CI: 3.3-10) for those aged 12 to 17 months (Table 3). The association for living in a household that owned livestock (versus living in a household that did not own livestock) was of borderline significance (odds ratio: 1.3; 95% CI: 1.0-1.7). The variables with a *p*-value below 0.2 included in the mixed regression model were the construction of a floor, the ownership of goods, domestic animals, livestock and the availability of toilets. An additional file shows the result of univariate analysis in more detail [see Additional file 1].

**Table 3:** **Associations of *Campylobacter *** **infection with age group during follow-up, mixed logistic regression model, Moramanga, 2010-2012**

**Confounding variables**		**Presence of *Campylobacter***		**Crude OR**	Adjusted OR*
		**Yes**	**No**		
Age group	< 6 months	16 (3.0)§	521 (97.0)§	Reference	Reference
	6-11 months	94 (13.1)	619 (86.9)	4.9 (2.7-8.9)†	5.0 (2.9-8.6)†
	12-17 months	90 (15.0)	509 (85.0)	6.0 (3.3-10.9)	5.7 (3.3-10.0)
	18-23 months	51 (9.0)	514 (91.0)	3.3 (1.8-5.9)	3.3 (1.8-5.8)
	24-29 months	25 (6.0)	396 (94.0)	2.2 (1.2-4.0)	2.1 (1.1-4.0)
	30-36 months	14 (4.9)	272 (95.1)	1.8 (0.8-3.9)	1.6 (0.8-3.4)
**Total**		**290**	**2831**		

†Numbers in brackets, 95% confidence interval; OR: odds ratio.

§Numbers in brackets, percentage.

*Adjusted for the ownership of livestock, mixed models were generated from 3121 observations.

Antimicrobial treatments were given to children with diarrhoea who had fever and to children with blood or mucus in their faeces. Antimicrobial treatment was given to 18.5% (85/459) of patients with diarrhoeal episodes due to fever and to 17.8% due to the presence of blood or mucus in the faeces (3.1% for blood and 14.8% for mucus in the faeces). Mild dehydration was observed in four episodes of diarrhoea.

We observed 475 diarrhoeal episodes with a median duration of 4.5 days (IQR: 5 days), resulting in an annual incidence rate of 0.7 episodes/child (95% CI: 0.7-0.8 episodes/child). We were able to collect stool samples for 96.6% (459/475) of all diarrhoeal episodes. Overall, 53.7% (*n* = 273) of the children presented at least one episode of diarrhoea during the observation period. The highest incidence rates were obtained for children under the age of 12 months. The annual incidence was 0.8 episodes/ child (95% CI: 0.7-0.9 episodes/child) in children aged 0 to 11 months and 0.5 episodes/child (95% CI: 0-0.6 episodes/child) for children over the age of 12 months.

The overall annual incidence of symptomatic *Campylobacter* infections was 0.05 episodes/child. The median duration of symptomatic *Campylobacter* infection was five days, with an IQR of 4.3 days. The mean age for symptomatic *Campylobacter* infection was 13.8 months (standard deviation: 7.1; range: 2.7-33.8 months; median: 11.8 months). Among the children with diarrhoea, 13.2% (36/273) excreted *Campylobacter* and 16.7% (6/36) received antimicrobial treatments. The frequency of symptomatic *Campylobacter* infections did not differ between age groups (*p* = 0.3, Wald *z*-statistic test).

*Campylobacter* was isolated from 201(39.6%) asymptomatic children. The mean age for asymptomatic *Campylobacter* infection was 14.7 months (standard deviation: 7; range: 1.3-35.2 months; median: 13 months).

There was a statistically significant association between *Campylobacter* primary infection and diarrhoea (*p* < 0.001, Wald *z*-statistics test). In the birth cohort of 64 children, the odds ratio for shedding *Campylobacter* for the first time among children with diarrhoea was 16.1 (95% CI: 1.8-140.8), taking children without diarrhoea as the reference group.

**Figure 1:**
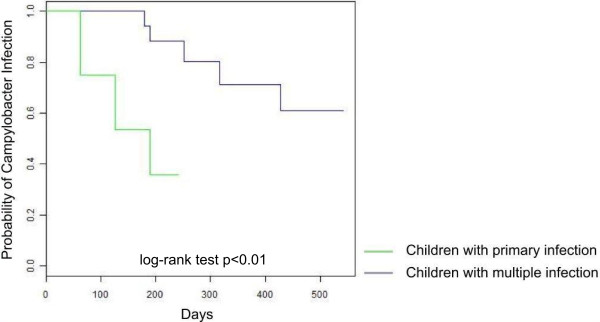
**Survival curves for *Campylobacter *** **infections in children with primary and multiple infections, Moramanga 2010-2012.**

Infection recurred more rapidly in children with primary infections than in those with multiple infections: 25% of children with primary infections had their second infection episode 63 days after the first episode, whereas the second episode occurred after 317 days after the first episode in children with multiple infections (*p* < 0.01, log-rank test). Figure 1 shows the survival curves for recurrent infection over time for the two groups. We found that children with primary infection living in a household that have mud flooring was 6.1 times at risk (hazard-ratio: 6.1, CI 95%: 1.4-25.5) to have recurrent infections more rapidly than those living in household with floor made with concrete materials (cement, tiles).

## Discussion

To our knowledge, this is the first cohort study on diarrhoea in children in Madagascar.

Our results provide an indication of the role of the immunity on the occurrence of *Campylobacter* infection in children in Moramanga, as we observed an age-related decrease in infection rates and the time to recurrence was shorter in children with primary infections than in those with multiple infections. Our data also suggest that the first *Campylobacter* infection was associated to diarrhoea.

We found an association between age group and infection, as reported in other developing countries [14,15], with infection rates increasing up to the age of six months and then decreasing after 30 months. In our cohort of children, immunity seemed to decrease the probability of acquiring infection in response to further exposures over time [16,17]. The shorter time to recurrence of infection in children with primary infection may reflect the immature and naive nature of the immune system in this group. The immune system is known to be immature at birth, with microbial colonization of the intestine playing a role in the maturation of the immune cell response [18,19]. Repeated exposures may be required to achieve immunity. Prior immunity has also been demonstrated to be important for protection against *Campylobacter* in other studies [20]. The first primary infection in our study occurred at the age of five months, suggesting a contribution of antibody transfer from the mother during the first five months of life. Susceptibility to *Campylobacter* infection in early infancy may thus be reduced by the acquisition of passive immunity from placentally transferred antibodies from immune mothers [17]. A previous study conducted in the Central African Republic also suggested a protective role for maternal antibodies. Children who had *Campylobacter* infections during the first six months of life had significantly fewer anti-flagellum antibodies at birth than those who did not have *Campylobacter* infections during this period [21,22]. Breastfeeding may also affect the development of immunity during early infancy; data about breastfeeding were not available during the follow-up period. We found that for children with primary infection, living in a household that have mud flooring is associated with an increased risk of having recurrent episodes. A mud flooring habitation may be considered as a proxy of a low socio-economic status of the household, human *Campylobacter* infection was already reported to be associated with poverty [15]. Our observation may also suggest that there is a risk of *Campylobacter* transmission in environments where there may be a poor hygiene conditions.

We conclude that the first *Campylobacter* infection was pathogenic. An analysis performed in a subset of the cohort, in newborns enrolled during the first 28 days of life, corroborated this finding. Reports of the pathogenicity of *Campylobacter* in different studies from around the developing world have not been consistent [7,8,15]. The development of diarrhoea during *Campylobacter* infection may depend on bacterial virulence and host susceptibility factors [23], and strains of *Campylobacter* may differ in pathogenicity. There may also be animal host-adapted genotypes that never or rarely cause diarrhoea in humans [24]. Despite the many studies carried out on *Campylobacter*, we still need to elucidate the mechanism by which *Campylobacter* causes diarrhoea in humans and its interaction with the human immune system.

Our data showed that species-specific immunity is less likely to occur, however it is difficult to conclude about species-specific resistance to reinfection as it is possible that there was a reinfection with the same species but with strains having different antigenic properties, thus immunity is not effective. Strain and serotype differences might have also an importance in protective immunity; immunity in human seems to be serotype-specific [25], neither genotypic nor serotyping analysis could not be performed in this study. In addition, host-specific intestinal microbiota composition may play also an important role in the proper development of immune system. Mechanisms underlying colonization resistance against *Campylobacter* infection are multifactorial. Repeated exposure is required for the generation of acquired immunity, but exposure to different strains of *Campylobacter* and overwhelming challenge may overcome immunity [21]. In developing countries, repeated exposure may subvert or suppress immune responses rather than leading to protection [26].

The rate of *Campylobacter* isolation in our study area was 9.3% and the annual incidence of symptomatic infection was 0.05 episodes/child. The rate of isolation of *Campylobacter* was lower than that reported for Peruvian children [15] but higher than that estimated for an Egyptian cohort [8]. However, the annual incidence of symptomatic infection in our study was only one tenth that reported in Peru [15], Mexico [7] and Egypt [8]. We documented *Campylobacter* in 13.2% of children with diarrhoea; this proportion has been estimated at 15.9% in the Central African Republic [21], 3.3% in Djibouti [27] and 0.8% in Guinea-Bissau [28]. These differences may reflect differences in environment, context and study design between settings. In addition, technical difficulties in the isolation of *Campylobacter* species in developing countries, due to their fastidious growth requirements and/or the relative insensitivity of culture techniques, may influence detection rates [29]. We do not have data on the presence of other bacterial or parasitic pathogens, thus we are therefore unable to ascertain which pathogen is the causative agent of the diarrhoeal episode. Assessing co-infections were of interest as the presence of any enteric pathogen may modulate the effect of other enteropathogens during concurrent infection to modify the clinical expression. During this study, stool samples have been stored for future testing.

In this study, we followed up a small sample of children included before the age of 28 days. An epidemiological study on this birth cohort might provide a better assessment of the role of immunity in determining the occurrence of infection.

We have not included in the logistic mixed model a random-effect that account for clustering through time. This could have an impact on our results. However, the individual random-effect might already account for this autocorrelation over time even if the time is not explicitly represented. Visits were made at regular intervals and we have not visited many children at very short intervals.

## Conclusion

This study suggests the role of prior immunity and repeated exposures on the occurrence of *Campylobacter* infection in children in the rural areas of Befotsy and Ampitambe, like in the other parts of the developing world. The association between infection and diarrhoea during the first year of life, the effect of the quality housing and the hygiene practices on the occurrence of recurrent episodes highlight the need for greater awareness of diarrhoeal disease prevention measures in infants and the importance of controlling *Campylobacter* infection. Prioritising interventions, such as those that may improve hygiene-related behaviors and/or the development of vaccines for prevalent *Campylobacter* serotypes, might have an impact on diarrhoeal illnesses in the developing world. The Moramanga HDSS constitutes a potential research platform for *Campylobacter* infection studies and vaccine trials.

## Competing interests

The authors have no competing interests to declare.

## Authors’ contributions

RVR conceived and co-ordinated the study, performed the statistical analysis and drafted the manuscript; FR participated in the design of the study and carried out the biological analysis of stool samples; PS participated in the design of the study and was involved in revising the manuscript critically for important intellectual content; HCR performed the PCR analysis; AR performed the field study and participated in the coordination of the study; IMR performed the PCR analysis; RR participated in the design of the study and the collection of data; VR participated in the design of the study and was involved in revising the manuscript critically for important intellectual content. All authors have read and approved the final manuscript.

## Pre-publication history

The pre-publication history for this paper can be accessed here:

http://www.biomedcentral.com/1471-2334/14/372/prepub

## Supplementary Material

Additional file 1 Univariate analysis of risk factors for Campylobacter infection, Moramanga, 2010-2012. Click here for file
