# Supplementary material for: Campylobacter infection in a cohort of rural children in Moramanga, Madagascar
Source: BMC Infect Dis. 2014 Jul 5;14:372. doi: 10.1186/1471-2334-14-372 (PMC4094412; doi:10.1186/1471-2334-14-372)
Supplement: Additional file 1 — Univariate analysis of risk factors for Campylobacter infection, Moramanga, 2010-2012. [file 1471-2334-14-372-S1.docx]

| **Characteristics**  Univariate analysis of risk factors for Campylobacter infection, Moramanga, 2010-2012 | **Campylobacter** | | **p** |
| --- | --- | --- | --- |
|  | Yes (%) | No (%) |  |
| Sex Male  Female | 163 (9.7)  156 (8.9) | 1518 (90.3)  1587 (91.1) | 0.4 |
| Age group (months)  < 6  6-11  12-17  18-23  24-29  30-36 | 18 (2.8)  109 (13.4)  100 (15.1)  52 (8.7)  26 (5.9)  14 (4.8) | 610 (97.2)  703 (22.6)  559 (84.9)  547 (91.3)  413 (94.1)  273 (95.2) | <0.01 |
| Underweight  Yes  No  Not available | 21 (9.3)  298 (9.3)  0 | 203 (90.7)  2898 (90.7)  4 | 0.9 |
| Stunting  Yes  No  Not available | 68 (10.3)  251 (9.0)  0 | 592 (89.7)  2509 (91.0)  4 | 0.4 |
| Wasting  Yes  No  Not available | 2 (6.0)  317 (9.3)  0 | 31 (94.0)  3070 (90.7)  4 | 0.7 |
| Source of lighting  Electricity  Candle/Petrol  No light  Not available | 0 (0.0)  284 (9.3)  6 (10.5)  29 | 14 (0.4)  2766 (97.7)  51 (89.5)  274 | 0.4 |
| Floor  Concrete  Mud  Not available | 179 (8.7)  111 (10.4)  29 | 1876 (91.3)  955 (89.6)  274 | 0.1* |
| Fuel for cooking  Purchased  Not purchased  Not available | 11 (7.9)  88 (8.1)  220 | 128 (92.1)  992 (91.9)  1985 | 0.9 |
| Goods  Yes  No  Not available | 283 (9.2)  7 (17.9)  29 | 2799 (90.8)  32 (82.1)  274 | 0.1* |
| Domestic animals  Yes  No  Not available | 98 (10.4)  192 (8.8)  29 | 844 (89.6)  1987 (91.2)  274 | 0.1* |
| Fowl  Yes  No  Not available | 187 (9.4)  103 (9.1)  29 | 1807 (90.6)  1024 (90.9)  274 | 0.8 |
| Livestock  Yes  No  Not available | 89 (11.3)  201 (8.6)  29 | 700 (88.7)  2131 (91.4)  274 | 0.03* |
| Garbage in the concession  Yes  No  Not available | 159 (9.9)  133 (8.9)  27 | 1443 (90.1)  1355 (91.1)  307 | 0.4 |
| Ownership of latrin  Yes  No  Not available | 172 (8.6)  118 (10.5)  29 | 1830 (92.4)  1001(89.5)  274 | 0.08* |
| Shower area  Yes  No  Not available | 34 (8.4)  255 (9.4)  30 | 369 (91.6)  2461 (90.6)  275 | 0.6 |
| Presence of a soap the day of interview  Yes  No  Not available | 196 (9.3)  92 (9.4)  31 | 1916 (90.7)  891 (90.6)  298 | 0.9 |
| Source of drinking water  Surface waters  Tubewell, borehole  Not available | 176 (9.6)  114 (8.9)  29 | 1659 (90.4)  1172 (91.1)  274 | 0.5 |
| Water drinking storage  Protected  Unprotected  Not available | 96 (10.3)  194 (8.9)  29 | 840 (89.7)  1991 (91.1)  274 | 0.2 |
| Availability of a cooking area  Yes  No  Not available | 267 (9.2)  23 (10.9)  29 | 2643 (90.8)  188 (89.1)  274 | 0.4 |
| Education level of the mother  Primary  Secondary  Superior  No education  Not available | 200 (9.6)  55 (8.3)  2 (15.4)  23 (12.0)  39 | 1895 (90.4)  603 (91.7)  11 (84.6)  168 (88.0)  428 | 0.4 |
| Age of the mother (mean ±SD) | 29 (9.8) | 28.2 (8.9) | 0.2 |
| Number of persons/room (mean ±SD) | 3.9 (1.8) | 3.9 (1.6) | 0.9 |

*SD : standard- deviation*
